# Supplementary material for: Clarifying Microbial Nitrous Oxide Reduction under Aerobic Conditions: Tolerant, Intolerant, and Sensitive
Source: Microbiol Spectr. 2023 Mar 16;11(2):e04709-22. doi: 10.1128/spectrum.04709-22 (PMC10100939; doi:10.1128/spectrum.04709-22)
Supplement: Supplemental file 1 — Supplemental material. Download spectrum.04709-22-s0001.pdf, PDF file, 5.6 MB [file spectrum.04709-22-s0001.pdf]

Supplementary Materials

Clarifying Microbial Nitrous Oxide Reduction Under Aerobic Conditions: Tolerant, Intolerant, And Sensitive

Zhiyue Wang[1], Nisha Vishwanathan[1], Sophie Kowaliczko[1], Satoshi Ishii[1,2,*]

[1] BioTechnology Institute, University of Minnesota, St. Paul, MN, USA

[2] Department of Soil, Water, and Climate, University of Minnesota, St. Paul, MN, USA

[*] Corresponding author, Email: ishi0040@umn.edu

Figure S1. Non-competitive inhibition model fitting to measured N_2O reduction rates of *Gemmatimonas aurantiaca* T-27 under varying N_2O and O_2 concentrations.

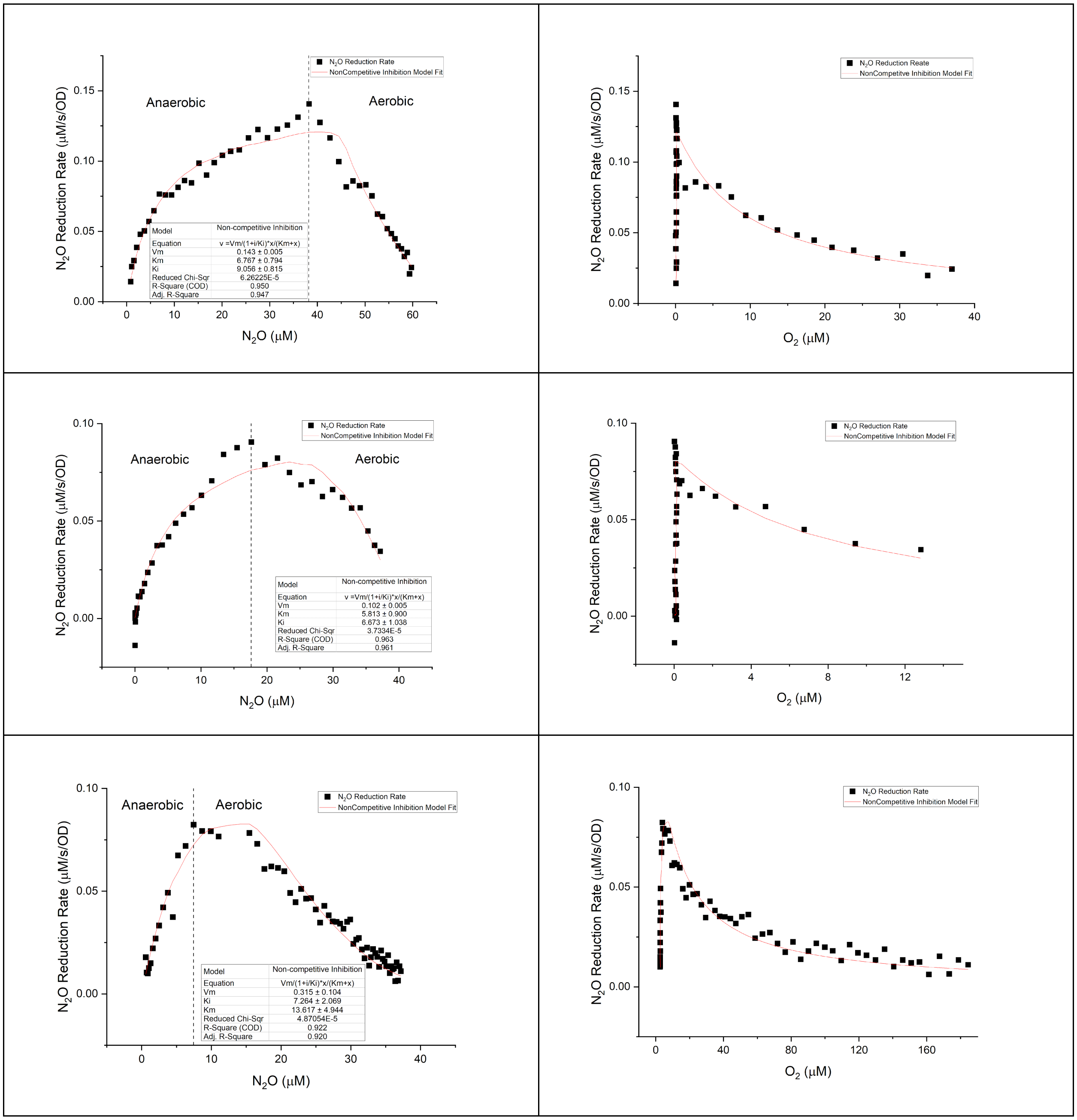

Figure S2. AlphaFold2 predicted protein models of Clade I and Clade II of nitrous oxide reductase from the following selected bacterial strains: (A) *Stutzerimonas stutzeri* ZoBell, (B) *Stutzerimonas stutzeri* TR2, (C) *Pseudomonas aeruginosa* PAO1, (D) *Paracoccus denitrificans* JCM 21484, (E) *Azospirillum brasilense* Sp7, (F) *Dechloromonas aromatica* RCB , and (G) *Gemmatimonas aurantiaca* T-27. Reaction centers of Cu_A (red) and Cu_Z (pink) are highlighted.

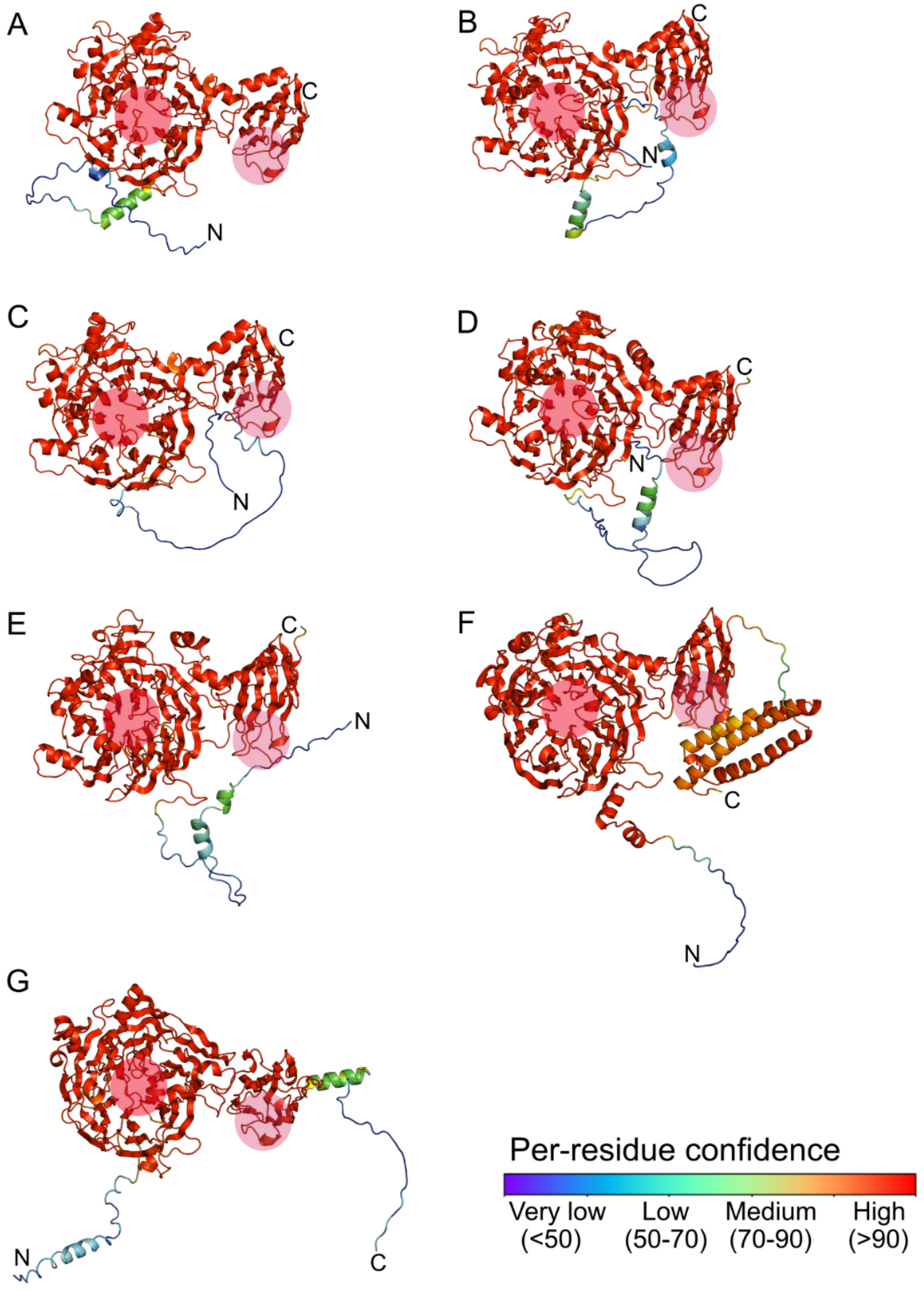

Figure S3. Multiple amino acid sequence alignment of NosZ from *S. stutzeri* ZoBell (Pst _1), *S. stutzeri* TR2 (Pst_2), *Ps. aeruginosa* PAO1 (Pae), *Pa. denitrificans* JCM 21484 (Pde), *A. brasilense* Sp7 (ABr), *D. aromatica* RCB (Dar), and *G. aurantiaca* T-27 (Gau).

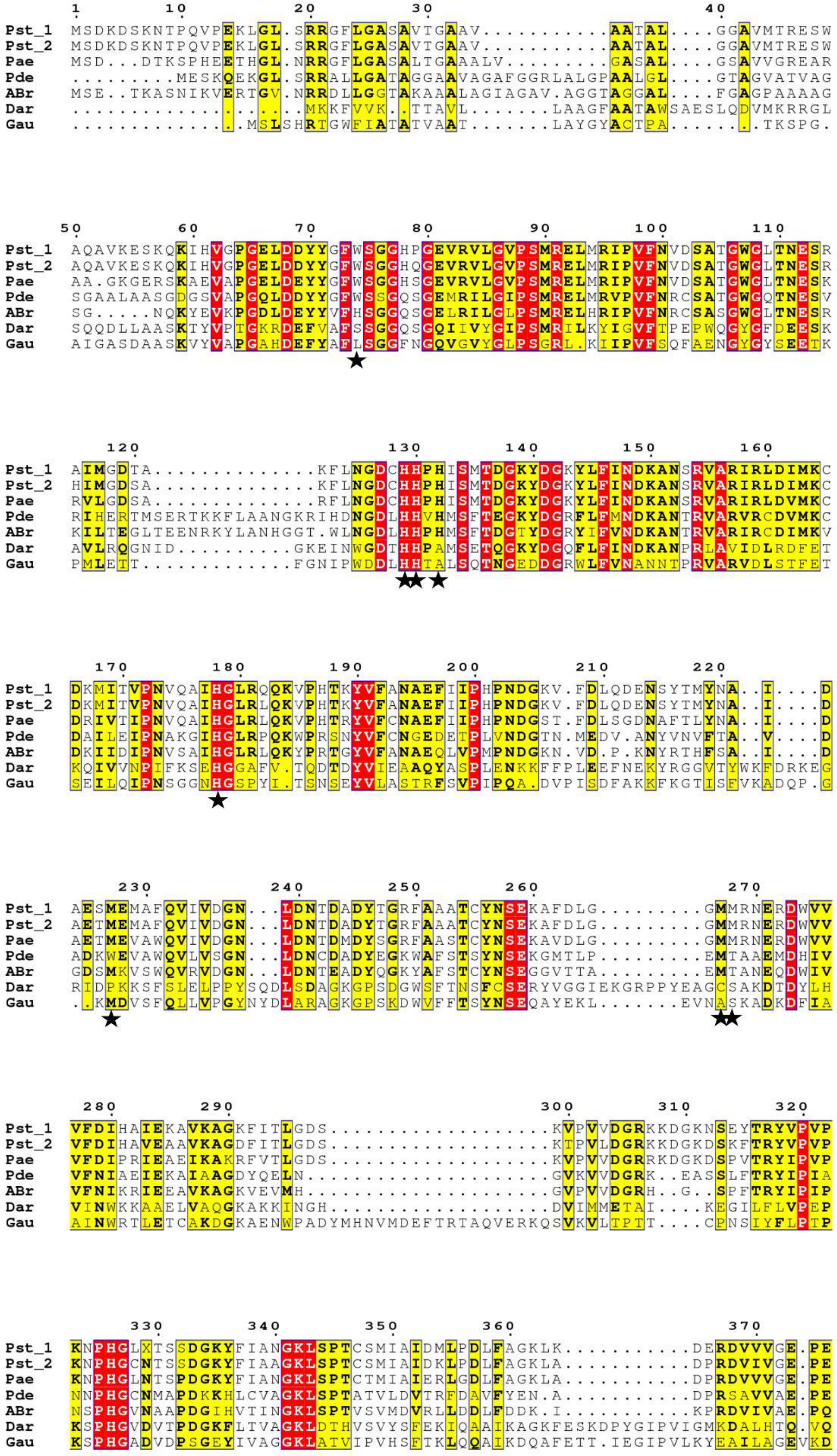

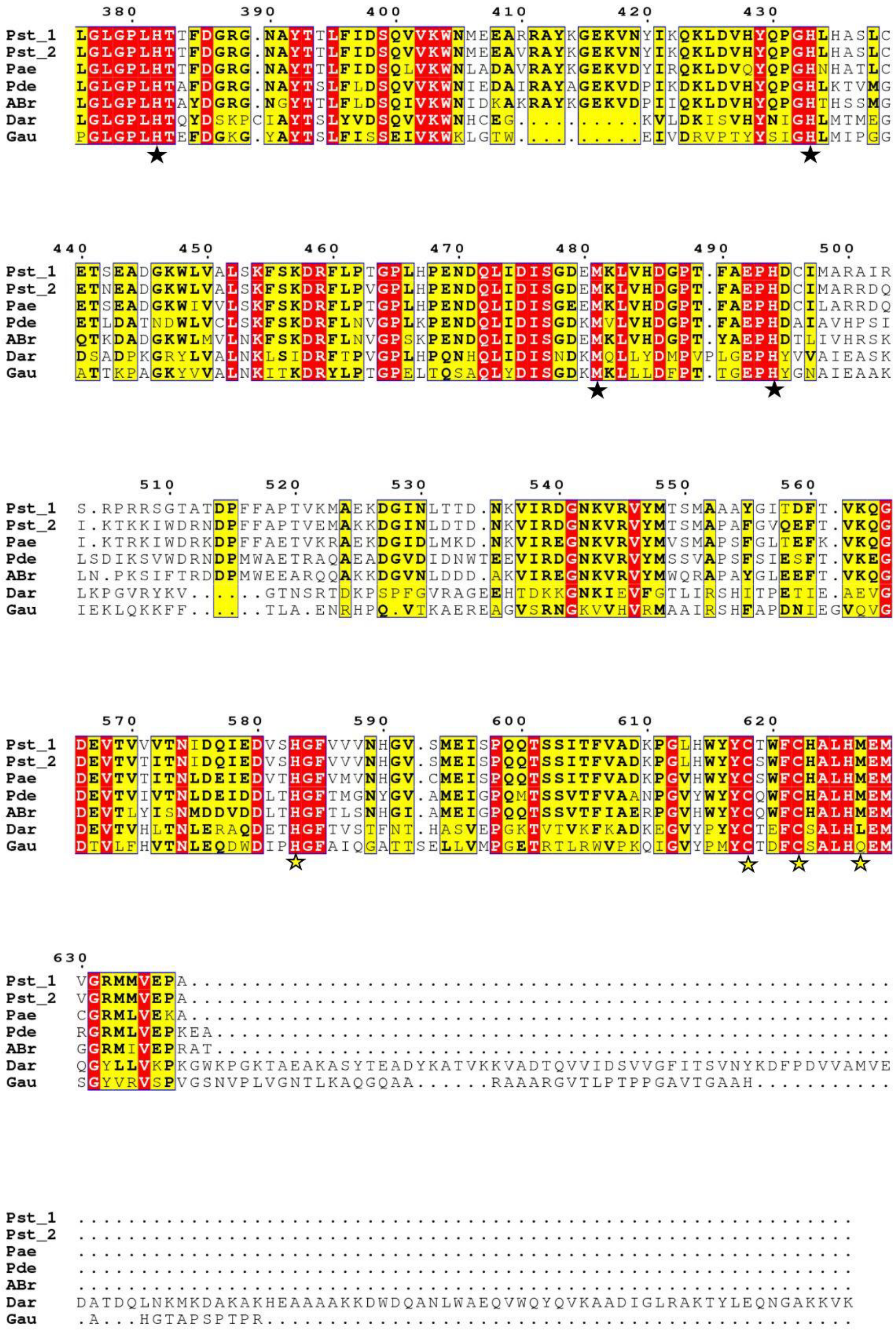
380 390 400 410 420 430
Pst_1 LGLGPLHTTFDGRG.NAYTTLFIDSQVVKWNMEEARRAYKGEKVNYIKQKLDVHYQPGHLHASLC
Pst_2 LGLGPLHTTFDGRG.NAYTTLFIDSQVVKWNMEEAVRAYKGEKVNYIKQKLDVHYQPGHLHASLC
Pae LGLGPLHTTFDGRG.NAYTTLFIDSQLVKWNLADAVRAYKGEKVDYIRQKLDVQYQPGHNHATLC
Pde LGLGPLHTAFDGRG.NAYTSLFLDSQVVKWNIEDAIRAYAGEKVDPIKDKLDVHYQPGHLKTVMG
ABr LGLGPLHTAYDGRG.NGYTTLFLDSQIVKWNIDKAKRAYKGEKVDPIIQKLDVHYQPGHTHSSMG
Dar LGLGPLHTQYDSKPCIAYTSLYVDSQVVKWNHCEG..........KVLDKISVHYNIGHLMTMEG
Gau PGLGPLHTEFDGKG.YAYTSLFISSEIVKWKLGTW..........EIVDRVPTYYSIGHLMIPGG
440 450 460 470 480 490 500
Pst_1 ETSEADGKWLVALSKFSKDRFLPTGPLHPENDQLIDISGDEMKLVHDGPT.FAEPHDCIMARAIR
Pst_2 ETNEADGKWLVALSKFSKDRFLPVGPLHPENDQLIDISGDEMKLVHDGPT.FAEPHDCIMARRDQ
Pae ETSEADGKWIVVLSKFSKDRFLPTGPLHPENDQLIDISGEEMKLVHDGPT.FAEPHDCILARRDQ
Pde ETLDATNDWLVCLSKFSKDRFLNVGPLKPENDQLIDISGDKMVLVHDGPT.FAEPHDAIAVHPSI
ABr QTKDADGKWLMVLNKFSKDRFLNVGPSKPENDQLIDISGDEMKLVHDGPT.YAEPHDTLIVHRSK
Dar DSADPKGRYLVALNKLSIDRFTPVGPLHPQNHQLIDISNDKMQLLYDMPVPLGEPHYVVAIEASK
Gau ATTKPAGKYVVALNKITKDRYLPTGPELTQSAQLYDISGDKMKLLDFPT.TGEPHYGNAIEAAK
510 520 530 540 550 560
Pst_1 S.RPRRSGTATDPFFAPTVKMAEKDGINLTTD.NKVIRDGNKVRVYMTSMAAAYGITDFT.VKQG
Pst_2 I.KTKKIWDRNDPFFAPTVEMAKKDGINLDTD.NKVIRDGNKVRVYMTSMAPAFGVQEFT.VKQG
Pae I.KTRKIWDRKDPFFAETVKRAEKDGIDLMKD.NKVIREGNKVRVYMVSMAPSFGLTEFK.VKQG
Pde LSDIKSVWDRNDPMWAETRAQAEADGVDIDNWTEEVIRDGNKVRVYMSSVAPSFSIESFT.VKEG
ABr LN.PKSIFTRDDPMWEEARQQAKKDGVNLDDD.AKVIREGNKVRVYMWQRAPAYGLEEFT.VKQG
Dar LKPGVRYKV......GTNSRTDKPSPFGVRAGEEHTDKKGNKIEVFGTLIRSHITPETIE.AEVG
Gau IEKLQKKFF......TLA.ENRHPQ.VTKAEREAGVSRNGKVVHVRMAAIRSHFAPDNIEGVQVG
570 580 590 600 610 620
Pst_1 DEVTVVVTNIDQIEDVSHGFVVVNHGV.SMEISPQQTSSITFVADKPGLHWYYCTWFCHALHMEM
Pst_2 DEVTVTITNIDQIEDVSHGFVVVNHGV.SMEISPQQTSSITFVADKPGLHWYYCSWFCHALHMEM
Pae DEVTVTITNLDEIEDVTHGFVMVNHGV.CMEISPQQTSSITFVADKPGVHWYYCSWFCHALHMEM
Pde DEVTVIVTNLDEIDDLTHGFTMGNYGV.AMEIGPQMTSSVTFVAANPGVYWYYCQWFCHALHMEM
ABr DEVTLYISNMDDVDDLTHGFTLSNHGI.AMEIGPQQTSSVTFIAERPGVHWYYCQWFCHALHMEM
Dar DEVTVHLTNLERAQDETHGFTVSTFNT.HASVEPGKTVTVKFKADKEGVYPYYCTEFCSALHLEM
Gau DTVLFHVTNLEQDWDIPHGFAIQGATTSELLVMPGETRTLRWVPKQIGVYPMYCTDFCSALHQEM
630
Pst_1 VGRMMVEPA..
Pst_2 VGRMMVEPA..
Pae CGRMLVEKA..
Pde RGRMLVEPKEA..
ABr GGRMIVEPRAT..
Dar QGYLLVKPKGWKPGKTAEAKASYTEADYKATVKKVADTQVVIDSVVGFITSVNYKDFPDVVAMVE
Gau SGYVRVSPVGSNVPLVGNTLKAQGQAA......RAAARGVTLPTPPGAVTGAAH...........
Pst_1 ....
Pst_2 ....
Pae ....
Pde ....
ABr ....
Dar DATDQLNKMKDAKAKHEAAAAKKDWDQANLWAEQVWQYQVKAADIGLRAKTYLEQNGAKKVK
Gau .A...HGTAPSPTPR..

Figure S4. Identity matrix of NosZ amino acid sequences.

**Pst_1**	100%						
**Pst_2**	92.63%	100%					
**Pae**	77.51%	79.71%	100%				
**Pde**	54.85%	55.79%	57.23%	100%			
**ABr**	58.15%	58.93%	59.27%	61.97%	100%		
**Dar**	30.09%	30.09%	29.4%	31.13%	30.6%	100%	
**Gau**	28.05%	27.58%	28.93%	28.06%	27.35%	37.28%	100%
	**Pst_1**	**Pst_2**	**Pae**	**Pde**	**ABr**	**Dar**	**Gau**

IDENTITY STATS

```
min      = 27.35
max      = 92.63
mean     = 46.8857142857143
stddev   = 19.8470687703628
```

Figure S5. Experimental setup for microsensor tests.

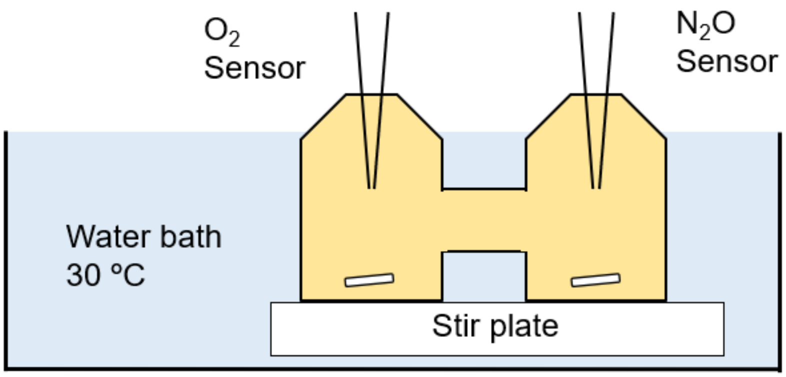

Table S1. Cross interferences of N_2O and O_2 on microsensors

Sample	Target N_2O (μM)	Target O_2 (μM)	N_2O Sensor Read (mV)	O_2 Sensor Read (mV)
Air (25°C)	0	236	5.5	153
10 mM PIPES buffer saturated with air (30°C)	0	236	7.5	163
10 mM PIPES buffer saturated with N_2 (30°C)	0	0	6.0	4.0
10 mM PIPES buffer saturated with 1.39% N_2O in N_2 (30°C)	300	0	565	5.0

Calculation for oxygen diffusion into cell membrane

Rate of O_2 respiration is

$$J_{resp} = \frac{R_{O2}}{A} = \frac{1\ \mu M/s/OD}{1 \times 10^9\ cell/OD \cdot mL\ \times\ 3.7 \times 10^{-8}\ cm^2/cell}$$
$$= 2.7 \times 10^{-2}\ mmol \cdot cm^{-2} \cdot s^{-1}$$

By Fick's first law, the concentration drop caused by respiration across cell membrane is

$$\Delta\varphi = \frac{J_{resp} \cdot \Delta x}{D} = \frac{2.7 \times 10^{-2}\ mmol \cdot cm^{-2} \cdot s^{-1}\ \times\ 5 \times 10^{-7}\ cm}{2 \times 10^{-5}\ cm^2 \cdot s^{-1}} = 0.7\ mM\ O_2$$

Assume the surface area a bacterial cell is similar to that of *E. coli*, which is 3.7 μm^2 (Levin & Angert, 2015), and the distance across a bilayer lipid membrane is about 5 nm (Clanton et al., 2013).

Reference

Clanton, T. L., Hogan, M. C., & Gladden, L. B. (2013). Regulation of Cellular Gas Exchange, Oxygen Sensing, and Metabolic Control. In *Comprehensive Physiology* (Vol. 3, pp. 1135–1190). Wiley. https://doi.org/10.1002/cphy.c120030

Levin, P. A., & Angert, E. R. (2015). Small but Mighty: Cell Size and Bacteria. *Cold Spring Harbor Perspectives in Biology*, *7*(7), a019216. https://doi.org/10.1101/cshperspect.a019216
